# Maintenance Debridement in Chronic Hard‐to‐Heal Wounds: Toward Dynamic Homeostasis and Precision Intervention

**DOI:** 10.1111/iwj.70930

**Published:** 2026-05-03

**Authors:** RuoDi Lu, Yi Zhang, Biao Cheng

**Affiliations:** ^1^ General Hospital of Southern Theater Command of PLA The First School of Clinical Medical University Guangzhou China; ^2^ Department of Neurology, Guangdong Neuroscience Institute, Guangdong Provincial People's Hospital (Guangdong Academy of Medical Sciences) Southern Medical University Guangzhou China; ^3^ Department of Burns and Plastic Surgery, General Hospital of Southern Theater Command of PLA Guangzhou China

## Abstract

Debridement is widely used across various wound types, but its biological significance differs fundamentally between acute and chronic hard‐to‐heal wounds. In acute wounds, debridement directly triggers the initiation of regeneration; however, in chronic wounds, persistent biofilm, insufficient angiogenesis and dysregulated inflammation lead to a prolonged cycle of inflammation and proliferation, making traditional one‐off debridement insufficient to change the healing trajectory. Therefore, the concept of debridement needs to evolve from an acute wound model to a sequential, biologically driven continuous management strategy. Maintenance debridement, by dynamically regulating the microenvironment, reducing pathological load and restoring a ‘healable state’, has emerged as the crucial bridge between inflammation control and regenerative therapies. This review systematically explains the theoretical basis and clinical value of maintenance debridement and explores the future direction of AI‐assisted precision debridement management.

## Highlights

- This review proposes the concept of “maintenance debridement” as a longitudinal management strategy aimed at achieving dynamic microenvironmental homeostasis in chronic wounds.
- It delineates the fundamental differences between acute and chronic wound debridement in terms of biological mechanisms and clinical objectives, particularly the interplay between pro‐healing drivers and pathological burdens.
- A conceptual framework is developed integrating positive reparative factors (e.g., oxygenation, cellular activity) and negative pathological factors (e.g., biofilm, inflammation, protease imbalance) within the wound microenvironment.
- Current evidence on debridement frequency is summarized, emphasizing individualized and continuous intervention strategies based on dynamic balancing of these factors.
- The potential role and current limitations of artificial intelligence in identifying microenvironmental dynamics and supporting precision decision‐making are critically discussed.

## 1 Introduction

### 1.1 Current Status and Clinical Challenges of Chronic Wounds

Chronic wounds represent a major and growing medical and economic burden worldwide. Their development is closely associated with chronic conditions such as diabetes, obesity and venous or arterial insufficiency, and their prevalence continues to increase in parallel with the rising burden of these underlying disorders. According to the *IDF Diabetes Atlas* (11th Edition), approximately 589 million adults aged 20–79 years were living with diabetes globally in 2024—roughly 1 in every 9 adults—and this number is projected to reach 853 million by 2050. Diabetes‐related mortality and healthcare expenditure are also substantial [1]. A systematic review reported a pooled prevalence of venous leg ulcers in the general population of approximately 0.32% (range: 0.12%–1.69%) [2]. Furthermore, a meta‐analysis including 39 studies and 2 579 049 hospitalized adults found a global prevalence of pressure injuries of 12.8% [3]. The five‐year mortality rate following lower‐extremity amputation in patients with diabetic foot ulcers has been estimated at 40%–70% [4].

Beyond physical morbidity, chronic wounds significantly impair health‐related quality of life and are closely associated with psychological comorbidities such as depression. Evidence suggests that depression is linked to delayed wound healing, increased complications and a higher risk of infection, highlighting the need to systematically integrate psychological assessment and intervention into wound management protocols [5]. Additional research has further demonstrated the persistent negative effects of chronic wounds on social participation and overall quality of life [6].

### 1.2 Pathophysiological Characteristics of Chronic Non‐Healing Wounds

Chronic wounds are characterized by impaired tissue perfusion, sustained hypoxia, biofilm formation and the accumulation of senescent cells—factors that collectively arrest the healing process in a prolonged inflammatory phase [7]. Unlike acute wounds, which can typically progress toward healing following a single thorough debridement, chronic wounds require repeated interventions to counteract recurrent biofilm formation and the persistent accumulation of inflammatory mediators such as matrix metalloproteinases (MMPs). Disrupting this vicious cycle is essential for re‐initiating the transition from the inflammatory to the proliferative phase [8]. Moreover, effective management of chronic wounds requires not only regular debridement to maintain a clean and physiologically favourable wound microenvironment, but also sustained support for regenerative processes to promote healthy tissue formation and advance wound repair [9].

Biofilm formation constitutes a critical barrier to healing; studies have demonstrated that biofilms markedly delay wound closure and increase the risk of infection [10]. The accumulation of senescent cells within chronic wounds further suppresses tissue regeneration and has emerged as an important therapeutic target in current research [11].

### 1.3 Mechanisms Underlying Delayed Wound Healing

Chronic non‐healing wounds arise from the interaction of multiple pathological processes, including persistent infection and biofilm formation, accumulation of senescent cells, impaired tissue perfusion and sustained inflammatory activation. Together, these factors disrupt the extracellular matrix, cellular migratory capacity, angiogenic support and reparative signalling required for progression from the inflammatory phase to the proliferative phase, thereby perpetuating non‐healing [10, 12, 13]. As these abnormalities persist, wounds may progressively enlarge and deepen, angiogenesis becomes further impaired, senescent cells continue to release pro‐inflammatory and matrix‐degrading mediators, and tissue architecture is progressively damaged. At the same time, biofilms mature and develop greater tolerance, further amplifying the infectious and inflammatory burden. This self‐reinforcing cycle contributes not only to wound progression, but also to substantial economic, physical and psychological burden for patients and their families [14, 15, 16].

**FIGURE 1:**
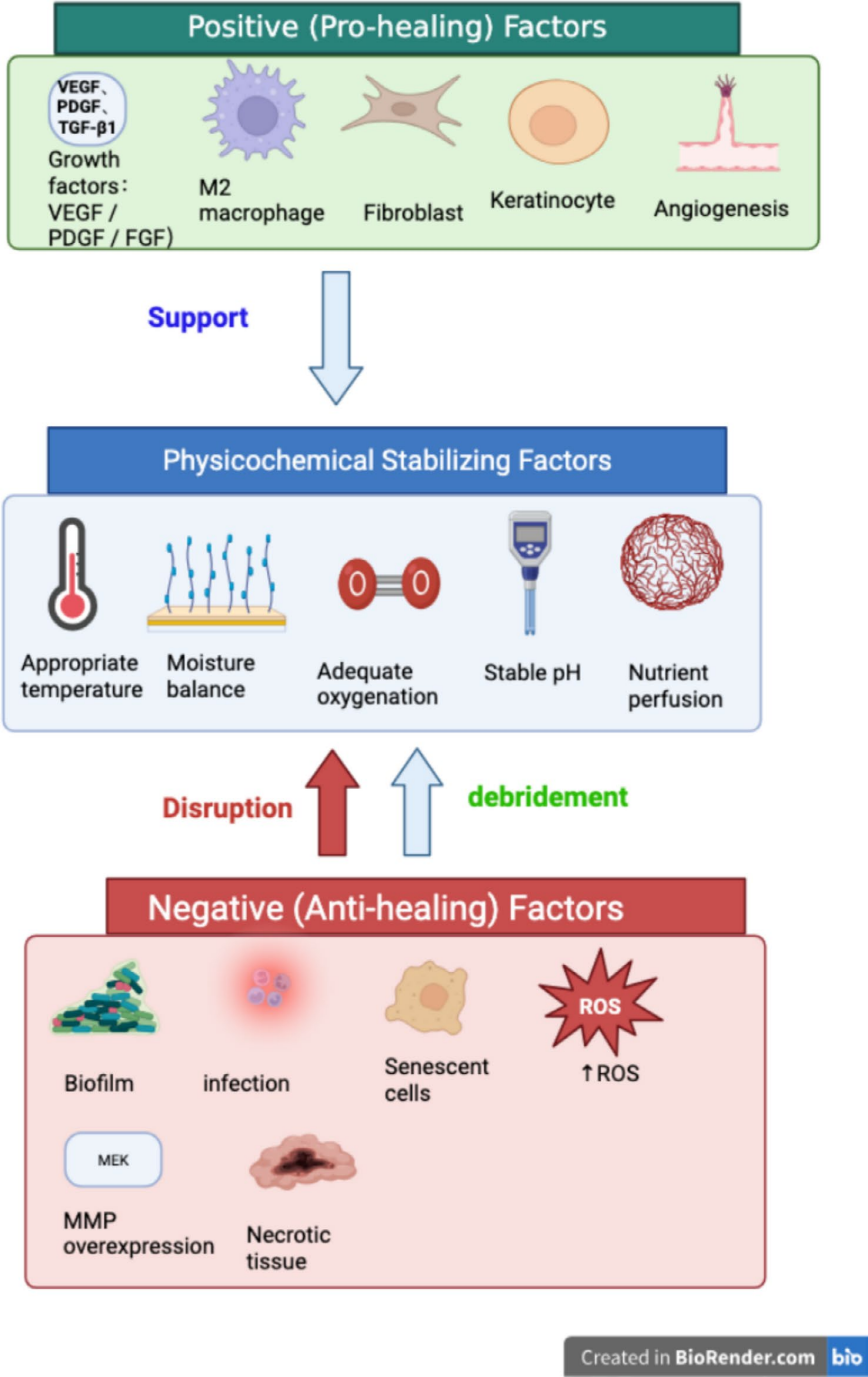
Schematic illustration of the major mechanisms underlying delayed healing in chronic hard‐to‐heal wounds.

For the purposes of this review, the principal mechanisms underlying delayed wound healing are organized into three interrelated domains: insufficient clearance dynamics, dysregulated regenerative dynamics and disrupted wound homeostasis, as illustrated in Figure 1. These categories are intended as a conceptual framework for discussion and do not represent mutually exclusive processes.

#### 1.3.1 Insufficient Clearance Dynamics

Effective management and clearance of detrimental wound components, including pathological wound exudate, biofilm and senescent or dysfunctional cells, are essential for successful wound repair.

##### 1.3.1.1 Wound Exudate

Both excessive and insufficient exudate can impair wound healing. The composition of wound exudate differs markedly between acute and chronic wounds. Acute wound exudate is rich in leukocytes and repair‐promoting factors, whereas chronic wound exudate contains elevated levels of proteases and pro‐inflammatory cytokines. In particular, matrix metalloproteinases (MMPs) are significantly upregulated, continuously degrading the extracellular matrix and inhibiting cell migration and granulation tissue formation. As healing progresses, these detrimental components typically decline [17, 18].

##### 1.3.1.2 Biofilm

Microbial proliferation is a critical step in the transition from acute to chronic wounds. Immune responses alone are often insufficient to effectively eradicate infection, allowing microorganisms to organize into single‐species or polymicrobial biofilms. Wound colonization involves a wide range of aerobic and anaerobic pathogens, including bacteria and yeast‐like fungi [19]. The predominant pathogens in chronic wounds include *Staphylococcus aureus* , *Pseudomonas aeruginosa* , β‐hemolytic *Streptococcus pyogenes* and *Candida albicans* [20, 21, 22]. These microorganisms form complex polymicrobial biofilms and secrete virulence factors and quorum‐sensing molecules, thereby significantly impairing immune clearance and tissue repair, and ultimately contributing to delayed healing and recurrent infection [23].

##### 1.3.1.3 Senescent and Dysfunctional Cells

Compared with acute wounds, chronic wounds frequently exhibit hallmarks of cellular senescence. Senescent cells display impaired proliferative and secretory functions and show reduced or absent responsiveness to normal healing signals [7]. Studies have shown that fibroblasts in venous ulcers and pressure injuries enter a senescent state, exhibit markedly reduced proliferative capacity and are directly associated with chronic non‐healing wounds [24, 25, 26]. Multiple senescent cell types—including keratinocytes, endothelial cells, fibroblasts and macrophages—can accumulate within chronic wounds [17, 27, 28, 29]. Mechanistically, cellular senescence is commonly associated with oxidative stress‐induced DNA damage, cell cycle arrest and metabolic abnormalities in patients with diabetes. These alterations may disrupt key intracellular pathways, such as the GSK‐3β/Fyn/Nrf2 signalling axis [28, 30].

Fibroblasts derived from chronic ulcers exhibit markedly reduced responsiveness to exogenous growth factors such as PDGF‐B and TGF‐β, a phenomenon that may be attributable to cellular senescence. Because chronic wounds accumulate large numbers of cells that are unable to respond to healing signals, the topical application of growth factors alone is often insufficient to achieve wound closure unless functionally competent neighbouring cells migrate into the wound bed [7].

In contrast to the approximately 6% prevalence of biofilm in acute wounds, biofilm formation in chronic wounds may reach up to 60%. Within these polymicrobial biofilms, interspecies interactions facilitate horizontal gene transfer and promote synergistic or competitive behaviors, thereby significantly enhancing tolerance to antimicrobial therapy [31].

#### 1.3.2 Dysregulated Regenerative Dynamics

In addition to impaired clearance capacity, chronic wounds also exhibit dysregulated regenerative dynamics. Many patients with chronic wounds have comorbidities such as diabetes, vascular disease, or age‐related metabolic decline, all of which limit the migration, proliferation and differentiation of fibroblasts, keratinocytes and endothelial cells [32]. Prolonged ischemia and oxidative stress further induce apoptosis and cellular senescence, resulting in reduced secretion of key growth factors—including VEGF, PDGF and TGF‐β1—and disruption of regeneration‐related signalling pathways [33].

Furthermore, the persistent inflammatory microenvironment is enriched in reactive oxygen species (ROS), proteases, pro‐inflammatory cytokines (such as TNF‐α and IL‐1β) and senescence‐associated secretory phenotype (SASP) factors. These mediators damage surrounding healthy tissue, destabilize the extracellular matrix and inhibit angiogenesis and collagen deposition [16, 34]. Collectively, these mechanisms hinder the orderly transition of repair from the inflammatory phase to granulation tissue formation and re‐epithelialization, ultimately leading to prolonged arrest of wound healing [13, 35].

#### 1.3.3 Disrupted Wound Homeostasis

Chronic wounds are characterized by persistent inflammation and infection, together with reduced efficiency of tissue renewal and repair. Over the prolonged disease course, recurrent ischemia–reperfusion injury, sustained hypoxia, oxidative stress and infectious stimuli can trigger widespread chronic inflammatory responses, placing the wound in a state of ‘high metabolic consumption but low repair efficiency’ [33, 36].

In this context, isolated or intermittent interventions are often insufficient to reverse the underlying pathology. Evidence indicates that the sustained presence of proteases, ROS and inflammatory mediators within the chronic inflammatory microenvironment continuously damages newly formed tissue and degrades extracellular matrix components, thereby offsetting the benefits of local treatment [37, 38]. Clinically, this is reflected in poor therapeutic outcomes and high recurrence rates. Mechanistic studies have further shown that these processes constitute key barriers to chronic wound healing. As the treatment course becomes prolonged, both patients and healthcare providers may develop reduced expectations regarding successful healing, further compounding the clinical challenge of hard‐to‐heal wounds.

## 2 The Role of Debridement in Chronic Wound Management: From Traditional Concepts to Maintenance Debridement

Traditional debridement concepts are primarily derived from experience in the management of acute wounds, in which the one‐time removal of necrotic tissue, foreign material and infectious foci can often restore the local conditions required for physiological healing. Such ‘one‐time, complete, surgical‐style’ debridement is therefore highly effective in many acute injuries. However, this model is frequently inadequate for chronic wounds, which are characterized by prolonged duration, persistent inflammation, recurrent infection, rapid biofilm re‐formation and sustained microenvironmental imbalance [39, 40] (Figure 1).

Figure created with BioRender.com. Published with permission under BioRender's Academic Licence. This figure is a conceptual model intended for schematic illustration of the relevant mechanisms and does not represent quantitative relationships derived from specific clinical data.

In this review, maintenance debridement is defined as a longitudinal, reassessment‐guided strategy involving repeated and relatively tissue‐preserving removal of necrotic burden and recurrent biofilm, with the aim of maintaining a wound microenvironment conducive to healing rather than achieving one‐time complete clearance. Against this background, maintenance debridement should be understood not simply as repeated tissue removal, but as a process‐oriented approach to continuous wound bed regulation throughout the course of treatment.

### 2.1 Conceptual Shift: From ‘Complete Removal’ to ‘Dynamic Control’

In acute wounds, debridement is primarily intended to remove local barriers to healing in a single or stage‐based intervention, thereby restoring the conditions necessary for physiological tissue repair. In chronic wounds, however, the objective is different. Because these wounds are characterized by recurrent biofilm formation, persistent inflammation and sustained bioburden, debridement is less a matter of achieving maximal removal in one session than of maintaining control over a continuously evolving pathological microenvironment [14, 41, 42] (Table 1).

Accordingly, the aim of maintenance debridement is not to achieve absolute cleanliness or complete sterility, but to keep the wound in a state more favourable for healing. Elevated bacterial burden has been associated with impaired healing, and a threshold of > 10^4^ CFU/g is often cited as a reference level suggesting the need for further intervention [43]. Likewise, because biofilm is highly prevalent in chronic wounds and may rapidly re‐form after debridement, repeated disruption of biofilm and reduction of local bioburden become central objectives of long‐term wound management [14, 41, 42]. In this sense, maintenance debridement represents a shift in emphasis—from one‐time complete removal to sustained dynamic control.

**TABLE 1:** Comparison of pathological characteristics and debridement strategies between acute and chronic wounds.

Feature	Acute wounds	Chronic wounds
Pathological/microenvironmental profile	Generally retain a relatively intact physiological healing trajectory, with orderly progression from inflammation to proliferation.	Commonly remain trapped in persistent pathological inflammation with necrotic tissue, biofilm, high bioburden, protease imbalance and wound‐edge stagnation.
Debridement goal	Remove local factors that prolong or amplify inflammation and support progression through the normal healing cascade.	Disrupt the vicious cycle of inflammation, biofilm, bioburden and tissue destruction to reconstruct a healable wound bed while preserving potentially salvageable tissue whenever possible.
Debridement approach/intensity	Often one‐time or stage‐based thorough debridement focused on foreign material, contaminants and clearly devitalized tissue.	Individualized and stratified debridement balancing removal of necrotic tissue and biofilm with preservation of tissue viability, perfusion and regenerative potential.
Debridement frequency	Usually once or repeated briefly according to injury stage and contamination/infection status.	Often requires regular, repeated and serial reassessment‐guided debridement.
Management concept	Procedural correction.	Dynamic long‐term management under the TIMERS framework.

*Note:* This table summarizes conceptual differences rather than standardized clinical protocols. These differences provide the conceptual basis for maintenance debridement in chronic wound care.

### 2.2 Mechanistic Shift: From ‘Decontamination’ to ‘Microenvironmental Remodelling’

Mechanistically, maintenance debridement aims to influence several wound microenvironmental parameters that are relevant to tissue repair. Through repeated, relatively atraumatic debridement, these parameters may be progressively improved and maintained within ranges more favorable for healing. However, they should not be regarded as rigid therapeutic endpoints of maintenance debridement; rather, they are better understood as reference indicators reflecting the state of the wound microenvironment.

#### 2.2.1 Bacterial Burden and Biofilm Control

Previous studies suggest that when bacterial burden falls below infection‐associated thresholds (approximately 10^4^–10^5^ CFU/g of tissue), the risk of clinical infection is relatively low. Similarly, limiting biofilm coverage to less than approximately 10% of the wound surface area has been associated with a reduced risk of inflammatory amplification [44]. Within this range, the host immune system may be better able to control residual pathogens without triggering overt inflammatory responses [43]. It should be noted, however, that these values are largely derived from experimental or observational studies and have not yet been established as universally accepted clinical criteria.

#### 2.2.2 Oxygenation Status (TcPO_2_)

Several studies have reported that TcPO_2_ ≥ 40 mmHg is generally associated with favourable healing potential, values of 30–40 mmHg may represent a relatively reparable range, and values < 30 mmHg are commonly indicative of inadequate perfusion [45]. It should be emphasized that these thresholds are used primarily for risk stratification and prognostic assessment; their value in guiding debridement frequency has not yet been prospectively validated.

#### 2.2.3 Wound pH

A pH range of 5.5–6.5 is considered favourable for keratinocyte migration and may inhibit the proliferation of certain bacterial species, whereas a pH > 7 is frequently associated with increased infectious or inflammatory activity [18]. Current evidence in this area is derived largely from experimental studies or small‐sample investigations, and its application to clinical decision‐making remains exploratory.

#### 2.2.4 Moisture Balance

Maintaining a moderately moist environment, such as a relative humidity of approximately 70%–90%, is generally considered beneficial for preserving cellular viability and supporting re‐epithelialization [29]. However, this range reflects general wound care principles and experimental observations rather than a rigorously validated universal clinical threshold.

#### 2.2.5 Protease Activity

The relative balance between matrix metalloproteinases (MMPs) and their tissue inhibitors (TIMPs) is considered relevant to the degree of matrix degradation and the wound's reparative capacity. Some studies have proposed that an MMP‐9/TIMP‐1 ratio of < 1 may indicate a relatively more favourable microenvironmental state [46]. Nevertheless, high‐quality clinical evidence supporting this ratio as a universally applicable intervention target remains lacking.

Overall, the parameters described above are best understood as reference ranges derived from existing evidence rather than as established universal clinical targets. These parameters are intended to inform physiological interpretation rather than prescribe fixed debridement targets in routine clinical practice. The use of physiological indicators to guide debridement strategies remains investigational, and its applicability across different wound types, as well as its clinical benefit, still requires confirmation through prospective studies.

### 2.3 Evolution From ‘Therapeutic Procedure’ to ‘Dynamic Management Strategy’

Within the TIMERS framework, debridement can be understood as a longitudinal component of chronic wound management rather than an isolated technical step. In this context, debridement is positioned at the starting point of the TIMERS model (Tissue, Infection/Inflammation, Moisture, Edge, Regeneration/Repair and Social factors) [47] and extends throughout the entire course of wound treatment. Maintenance debridement is not merely the removal of devitalized tissue; through periodic and controlled interventions, it helps maintain a manageable bacterial burden, appropriate moisture levels and adequate oxygenation, thereby providing a stable foundation for regenerative therapies such as stem cell‐based treatments, platelet‐rich plasma (PRP) and growth factors [48]. In this sense, maintenance debridement functions as a coordinating intervention that links wound bed preparation with subsequent regenerative support.

## 3 Maintenance Debridement: Frequency, Technique and Future Directions

Within the management framework described above, the clinical focus shifts to how maintenance debridement should be implemented—namely, when to debride, which method to employ and how to adjust debridement frequency and intensity according to wound type. In recent years, international research has progressively shifted its focus from whether debridement is necessary to more refined and quantifiable questions: when to debride, how much to debride and how to debride. This marks an important evolution toward precision medicine and data‐driven wound care.

### 3.1 Evidence‐Based Guidance on Debridement Frequency and Technique

Multiple large retrospective studies and several randomized controlled trials suggest a positive association between debridement frequency and wound healing rate. In a landmark analysis, Wilcox et al. examined 312 744 chronic wounds in 154 644 patients and found that more frequent debridement was associated with significantly faster healing, whereas debridement intervals longer than 14 days were associated with prolonged healing time [49]. Similarly, a prospective study showed that regular debridement every 7–14 days, compared with intermittent debridement, significantly reduced infection rates and shortened time to healing [41]. In a randomized controlled trial involving diabetic foot ulcers (DFUs), weekly versus biweekly sharp debridement did not result in a significant difference in complete healing rates; however, the weekly debridement group exhibited fewer infection recurrences and less biofilm re‐formation [39, 50]. Another randomized study evaluating ultrasound‐assisted debridement showed that weekly treatment, compared with biweekly treatment, improved granulation tissue formation and provided better exudate control [51]. When debridement intervals exceed 14 days, biofilm and microbial communities tend to re‐establish, inflammatory mediators such as MMP‐9 and TNF‐α may rise again, and local tissue oxygen tension (pO_2_) may fall below 30 mmHg, thereby contributing to delayed healing or even recurrent necrosis [42].

Nevertheless, debridement strategies must be tailored to the characteristics of different chronic wound types. Patients with diabetic foot ulcers frequently present with microcirculatory impairment and elevated infection risk, typically requiring sharp debridement combined with mechanical or enzymatic adjuncts, followed by regular reassessment after infection control. Venous leg ulcers are more commonly managed with mechanical or autolytic debridement in combination with compression therapy to reduce exudate, edema and pain. In contrast, patients with arterial ulcers or pressure injuries require individualized assessment of perfusion status. Excessive sharp debridement in the setting of marginal tissue hypoperfusion may further damage the wound edge; therefore, aggressive debridement should be avoided, and autolytic or enzymatic methods are generally preferred to maintain microenvironmental stability [39, 52, 53].

Taken together, existing studies suggest that regular, continuous and individualized debridement may be more important than simply increasing debridement frequency or depth in chronic wound management.

### 3.2 Levels and Limitations of Existing Evidence

Although several studies suggest that debridement frequency may be associated with the rate of chronic wound healing, the current evidence base remains subject to important limitations. First, there is substantial heterogeneity across studies with respect to wound type, patient comorbidities, vascular status and local perfusion, making direct comparison between findings difficult. Chronic wounds are inherently heterogeneous: diabetic foot ulcers, venous leg ulcers and pressure injuries differ considerably in pathophysiology, progression and therapeutic strategy, which complicates the development of uniform standards for debridement frequency.

Among the most widely cited studies is the retrospective analysis by Wilcox et al. which included 154 644 patients and 312 744 chronic wounds and found that more frequent debridement was associated with shorter time to healing [49]. However, this association should not be interpreted as proof of a direct causal relationship. Potential confounding factors—such as treatment adherence, frequency of clinical follow‐up, access to specialist care and overall quality of wound management—may simultaneously influence both debridement frequency and healing outcomes.

Furthermore, randomized controlled trials in this field remain limited in number and scale. For example, one randomized study in patients with diabetic foot ulcers comparing weekly sharp debridement with biweekly debridement found no statistically significant difference in complete healing rates between groups, suggesting that the effect of debridement frequency on healing outcomes is likely to be modified by multiple clinical and biological factors [50].

At the same time, most existing studies continue to rely on clinical endpoints such as wound area reduction or time to closure. These outcomes, although clinically relevant, may not adequately capture intermediate biological changes within the wound microenvironment. Large‐scale clinical trials incorporating physiological or biomarker‐based endpoints—such as bacterial burden, biofilm dynamics, local tissue oxygenation, pH, or protease activity—remain scarce. Recent studies have highlighted the close relationship between chronic wound microbial community structure, biofilm formation and healing progression; however, much of this evidence remains observational or experimental, and its direct incorporation into debridement‐guided clinical protocols has yet to be validated [12].

**FIGURE 2:**
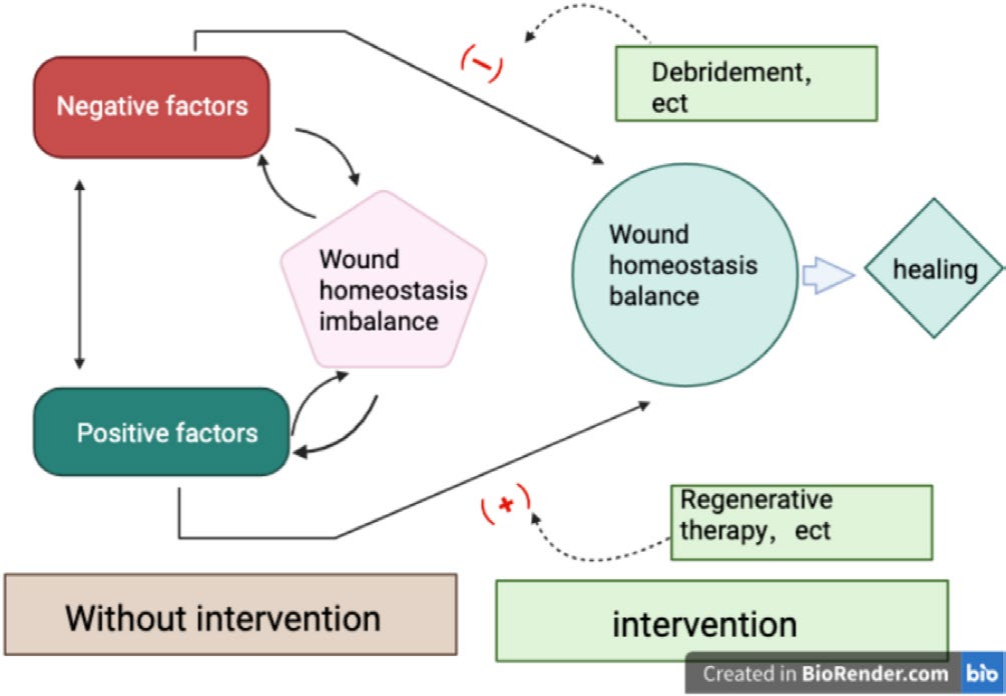
Conceptual framework of maintenance debridement and microenvironmental regulation in chronic wounds. Figure created with BioRender.com. Published with permission under BioRender's Academic Licence.

In summary, the available evidence broadly supports the importance of regular debridement in chronic wound management. Nevertheless, high‐quality prospective studies addressing optimal debridement frequency for specific wound types, verifiable physiological thresholds and standardized microenvironmental outcome frameworks remain limited. These issues require clarification through future large‐scale clinical trials with more rigorous and biologically informed study designs (Figure 2).

This figure is intended for schematic illustration only and does not represent a standardized workflow validated by clinical evidence.

## 4 Applications and Prospects of Artificial Intelligence in the Individualized Management of Chronic Wounds

With the rapid development of artificial intelligence (AI) and deep learning technologies, data‐driven approaches to assessment and decision support in chronic wound management have attracted increasing interest. Although conventional debridement can improve the wound microenvironment, decisions regarding timing, frequency and technique still depend largely on clinical experience. In recent years, convolutional neural network (CNN)‐based algorithms have been increasingly applied to wound image analysis. These methods can assist in automated wound boundary identification, tissue classification and prediction of healing trajectories, thereby providing quantitative support for wound assessment [54, 55].

In parallel, multimodal AI approaches integrating wound image analysis with sensor‐derived data—such as pH, temperature, humidity and local oxygen tension—are being explored for continuous wound monitoring and early detection of infection risk [56, 57]. In addition, some digital approaches have attempted to combine imaging, clinical and histopathological information in order to support wound evaluation and treatment planning [58, 59].

However, the application of AI in chronic wound management remains at an early stage of development. Most existing studies are based on retrospective datasets, proof‐of‐concept systems, or small validation cohorts and large prospective clinical studies remain lacking. In addition, challenges related to data standardization, model generalizability, algorithmic bias, interpretability, regulatory evaluation and workflow integration continue to limit clinical translation. Accordingly, AI should currently be regarded as an adjunctive investigational tool rather than a mature clinical system for guiding debridement decisions.

Future research should focus on multicenter data integration, standardized data collection, explainable model development, external validation, regulatory evaluation and clinically feasible translation pathways. If these challenges can be addressed, AI may eventually support more individualized, dynamic and data‐informed wound management strategies.

## 5 Conclusion

Emerging evidence suggests that maintenance debridement may serve as a continuous and potentially quantifiable strategy for regulating bacterial burden, modulating inflammation and maintaining physicochemical conditions within a range more favorable for tissue repair. By integrating the principles of wound bed preparation with dynamic microenvironmental management, maintenance‐oriented interventions shift chronic wound care from episodic procedural treatment toward sustained biological management.

At the same time, important clinical questions remain unresolved. Decisions regarding debridement frequency, technique selection and timing still rely on limited evidence and current studies are constrained by heterogeneity in wound type, insufficient physiological endpoints and the lack of standardized biomarker‐guided frameworks. Although artificial intelligence may offer a future avenue for real‐time assessment, trajectory prediction and data‐informed optimization of intervention timing, its application in debridement decision‐making remains investigational.

Overall, maintenance‐oriented wound management provides a clinically meaningful conceptual framework that integrates microenvironmental regulation, clearance dynamics and regenerative support. This approach holds substantial promise for improving healing outcomes, but its clinical value still requires further validation through high‐quality prospective studies and biologically informed precision‐management strategies.

## Funding

The authors have nothing to report.

## Ethics Statement

The authors have nothing to report.

## Conflicts of Interest

The authors declare no conflicts of interest.

## Data Availability

Data sharing not applicable to this article as no datasets were generated or analysed during the current study.
